# A quality improvement initiative for COPD patients: A cost analysis

**DOI:** 10.1371/journal.pone.0235040

**Published:** 2020-07-06

**Authors:** David Trout, Archita H. Bhansali, Dushon D. Riley, Fred W. Peyerl, Teofilo L. Lee-Chiong

**Affiliations:** 1 Philips, Sleep & Respiratory Care, Pittsburgh, PA, United States of America; 2 Boston Strategic Partners, Inc., Boston, MA, United States of America; 3 Department of Medicine, Division of Pulmonary, Critical Care & Sleep Medicine, Section of Sleep Medicine, National Jewish Health, Denver, CO, United States of America; Universite de Bretagne Occidentale, FRANCE

## Abstract

The objective of this analysis was to evaluate and report on the economic impact of implementing an integrated, quality, and operational improvement program on chronic obstructive pulmonary disease (COPD) care from acute through post-acute care settings. This initiative was established in a cohort of 12 hospitals in Alabama and sought to address COPD readmission through improved workflows pertaining to early diagnosis, efficient care transitions, and patient visibility across the entire care episode. Implementation of the initiative was influenced by lean principles, particularly cross-functional agreement of workflows to improve COPD care delivery and outcomes. A budget impact model was developed to calculate cost savings directly from objective data collected during this initiative. The model estimated payer annual savings over 5 years. Patients were classified for analysis based on whether or not they received noninvasive ventilation. Scenario analyses calculated savings for payers covering different COPD cohort sizes. The base case revealed annual per patient savings of $11,263 for patients treated through the quality improvement program versus traditional care. The model projected cumulative savings of $52 million over a 5-year period. Clinical incorporation of non-invasive ventilation (NIV) resulted in $20,535 annual savings per patient and projected $91 million over 5 years. We conclude that an integrated management program for COPD patients across the care continuum is associated with substantial cost savings and significantly reduced hospital readmissions.

## Introduction

Chronic obstructive pulmonary disease (COPD) is the fourth leading cause of death in the world [1]. A large part of the COPD burden of illness is due to disease exacerbations that require hospital admissions [2, 3], which can cost over $40,000 USD per hospitalization [3]. Recurrent hospitalizations for COPD patients may indicate poor long-term outcomes, such as end-stage lung disease, where hospital readmissions may not be in the best interest of the patient. Thus, implementing innovative measures that can reduce hospitalizations among patients with COPD represents an important objective. Coordinated care over the entire continuum has been proposed as a potential solution for improving care of patients with COPD, [4, 5], but specific strategies and end-to-end implementation workflows are not readily available or widely adopted.

Common COPD comorbidities, such as cardiovascular disease, heart failure, hypertension, and asthma, can mimic COPD symptoms. Studies have shown that adherence to Global Initiative for Chronic Obstructive Lung Disease (GOLD) guidelines for COPD diagnosis, especially the use of spirometry, has been suboptimal [6, 7]. Consequently, diagnosis of patients with diseases comorbid with COPD may be incomprehensive [8, 9] resulting in patients progressing to more severe stages of COPD before appropriate therapies can be implemented.

A fragmented healthcare system and inefficient allocation of resources can lead to avoidable hospital costs amounting to as much as $44 billion per year in the United States [10]. While this is a multifactorial phenomenon, one factor is inefficient transitions of care, or the movement of patients between health care practitioners and care settings as their condition and needs change.

There is a consensus among expert post-acute care professionals that a comprehensive end-to-end COPD care action plan can reduce COPD patient hospitalizations [4]. Economic modeling of outcomes from a quality improvement initiative for severe COPD patients has demonstrated that a multifaceted intervention program, including confirmatory diagnosis, and the appropriate use of home noninvasive ventilation (NIV), reduced readmissions within 30-days and its associated costs [11].

In Alabama, a large hospital was dissatisfied with the rate of readmissions for their COPD patients. The Vice President of Operations and Post-Acute Care services approached their home medical equipment provider (HME, Med-South Inc. AL) to discuss strategies to address this pain point. Given the possibility that other hospitals they partner with for home medical equipment services may also be experiencing high COPD readmissions, they approached their service network to determine if there were additional interested hospitals. The resulting joint venture included 12 Alabama hospitals, a recognized medical devices company, a third-party information technology specialist, and a home medical equipment (HME) provider.

The objectives of the venture were to optimize COPD management and outcomes by integrating the patient care pathway across acute hospital care through post-acute care management. Cross-functional identification of gaps in the care continuum and care transitions provided opportunities to reorganize and integrate already existing workflows and treatment paradigms to create consistent COPD case management and reduce readmissions. This end-to-end process addressed four main pillars of COPD management: 1) immediate risk assessment and diagnosis of disease, 2) systematic use of evidence-based guidelines for diagnosis and treatment, 3) efficient transitions and improved visibility from acute through post-acute care, and 4) systematic collection and integration of post-acute patient data into the centralized patient electronic health record (EHR).

This is not the first time the benefits of integrated care for COPD patients has been proposed [4, 5]. Several regulatory measures supporting the transition towards integrated care have been enacted in the U.S., including the IMPACT Act [12], and various CMS mandates [13–15], indicating that there is a desire to explore integrated care throughout US healthcare. To date however, there have been few comprehensive attempts to implement such programs.

Implementing this pathway required cross-functional agreement and buy-in by all care providers and IT stakeholders. Importantly, this initiative was not designed to influence hospital policies, protocols, or physician behavior; all final decisions regarding procedures, tests, interventions, and therapies if needed, were made by the patient’s physician.

Given previous evidence that a comprehensive, integrated and well-coordinated end-to-end patient management pathway may improve clinical outcomes while reducing cost [11] hospitalizations and generate cost savings to the payer, an economic model was developed to assess the economic impact of an Integrated Care Pathway (ICP) for the treatment of patients with COPD.

## Methods

### The Integrated Care Pathway initiative

**Fig 1:**
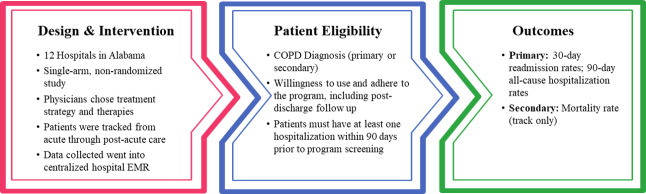
The Integrated Care Pathway (ICP). A schematic overview of the quality improvement initiative from which clinical event rates have been derived for the budget impact analysis.

See Fig 1 for an overview of the Integrated Care Pathway. The initiative was built upon lean-like premises seeking to improve efficiency and hospitalization rates through strategic workflow changes. A 3^rd^-party firm, IncreMedical®, engaged with hospital leadership and their HME partner through intensive cross-functional and cross-department (i.e. case management, internal, intensive, and emergency medicine, and information technology) discovery, followed by mutual adoption and agreement, order set creation, implementation, post-discharge data collection and transmission that was entered by the post-acute respiratory therapists into the hospital EHR, and data aggregation and analysis based on event rates.

Patients were deemed eligible for this program when, upon arrival at the hospital they exhibited any respiratory symptoms (e.g. coughing, labored breathing) or if any respiratory concerns stated by the patient. In total, 1,931 patients were screened for COPD. From that screened cohort, 53.5% (1,033) were diagnosed with COPD. Identification of previously undiagnosed COPD in patients with related co-morbidities, including, but not limited to, hypertension, pneumonia, heart failure, asthma, obesity, and sleep disturbances [16, 17]. These patients were screened using protocols and order sets that were established as part of the program.

Participating care providers had access to order sets created specifically by cross-functional teams during this initiative to support patient care covering, diagnostic laboratory tests, suggested consults, medications and other interventions, psychosocial support, and post-discharge transitioning, monitoring, and data reporting. Physician judgement was not influenced nor overridden to serve the initiative. For this initiative, all patients were discharged to home care and post-acute services (PAC) services were provided by the joint partner HME provider company (Med-South, Inc., Birmingham, AL) after the patient was discharged from the hospital and returned home. While providing home care, all patient data was collected by the HME RT on a secure mobile device, securely transmitted to and stored on a protected, third-party platform (MedAdept, IncreMedical®), and then transferred into the hospital EHR.

### Model perspective and analytic framework

A payer-perspective budget impact model was constructed using Microsoft® Excel. The budget impact model directly compared objective, outcome-driven cost savings associated with ICP implementation versus standard COPD care (i.e., ICP vs. 90-day pre-ICP standard COPD care). The model simulates annual, as well as 5-year cumulative, savings associated with all-cause, rolling 90-day hospitalizations.

### Model inputs and data sources

**Fig 2:**
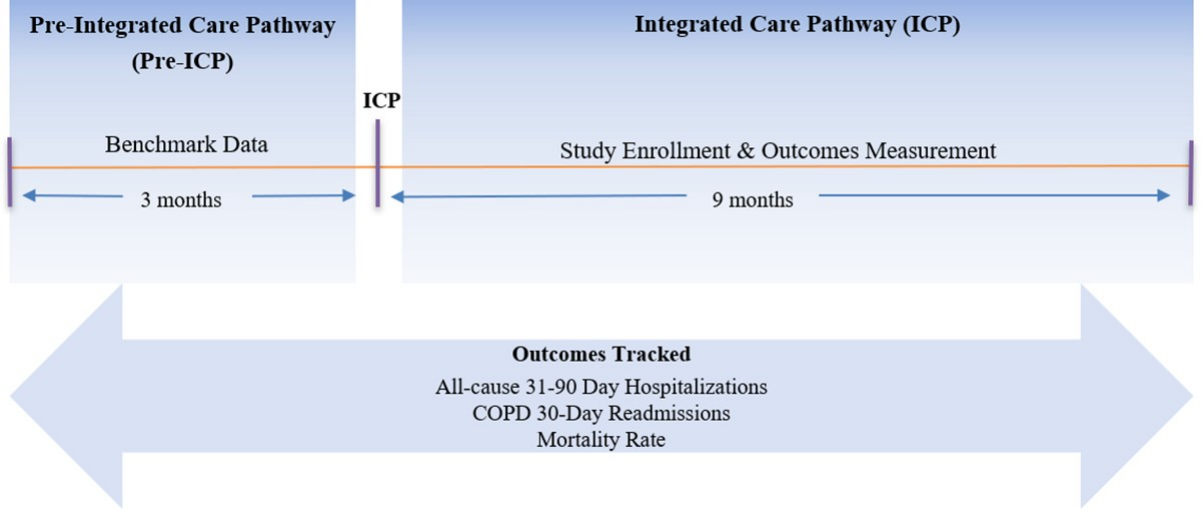
Integrated Care Pathway (ICP) time series event data collection. Schematic of time series data collection for COPD patients that enrolled in the ICP quality improvement program. Over the course of 9 months, rolling 90-day event data was tracked for enrolled patients. Event data for the 90-day period preceding enrollment in the ICP (Pre-ICP) is used as the traditional care comparative benchmark.

Deidentified patient outcome event rate data from the ICP was made available for the creation of the budget impact model. All patient event rates were derived from this quality improvement initiative exploring the clinical and economic outcomes of an end-to-end patient management program (see Table 1 for patient characteristics). Only patients who had at least 1) one hospitalization in the 90 days preceding entry into the ICP program and 2) 90 days of data in the ICP program were included in this analysis (Fig 2).

**Table 1:** Demographic and coverage breakdown for analyzed cohort (n = 1,033).

**Gender**	
Female	57%
Male	43%
**Age**	**Count**
20–29	6
30–39	7
40–49	20
50–59	146
60–69	298
70–79	383
80–89	150
90–99	28
**Payer Mix**	
Commercial	36.2%
Medicare	48.9%
Medicaid	1.3%
Self	11.8%
Other	1.7%
**Post-acute care Non-invasive Ventilation (NIV)**	
Not Prescribed	55%
Prescribed	45%

See Table 1 for a presentation of the model inputs. Event frequencies for patient readmission and hospitalization events were then derived from a 12-month window of this quality improvement initiative. NIV was provided by a home medical equipment provider if the physician determined that post-acute care ventilation was indicated as part of the ICP in the context of GOLD grading and other evidence-based criteria (e.g., fully-documented symptoms of hypoventilation, hypercapnia and/or hypoxemia during sleep) [18, 19].

Hospital admission costs were obtained from previously published data [20]. Reimbursements for hospital admission were defined by the Center for Medicare & Medicaid Services (CMS) reported rates (current as of August 2018) [21]. Device and supplies reimbursement were defined by 2019 CMS-reported rates [22].

### Analysis and outputs

Annual hospitalizations were calculated from the initial number of patients enrolled in the ICP program (year 0) or the number of surviving patients from the previous year (years 1–5) and hospitalization event frequency (Pre-ICP (standard care), ICP, or +NIV). The number of surviving patients in year 1 for each comparator was based on initial numbers of patients enrolled in the ICP x 1-year mortality rate. The number of surviving patients in subsequent years was calculated by applying the 1-year mortality rate to the number of patients surviving the preceding year. This approach led to years 2–5 patient populations. Total annual cost was calculated as the number of hospitalizations x reimbursement per hospitalization. See Tables 2 and 3 for complete list of defined calculations and base case values, respectively.

**Table 2:** Budget impact model definitions.

	Term	Method
**A**	Integrated Care Pathway (ICP)	An integrated care pathway was created over an 18-month period consisting of: • Customized, evidence-based management protocols (e.g., Global Initiative for Chronic Obstructive Lung Disease (GOLD), COPD Foundation) • Customized clinical pathways • Multi-faceted care management including customized transition from acute to post-acute care units Patient outcomes monitored and tracked included: • Hospital readmissions within 30 days of initial COPD admission (30-day Readmissions) • All-cause Hospitalizations between 31–90 days (31–90 Day Hospitalizations)
**B**	Patients who "drop out"	Only patients who met the inclusion criteria described above [A] were included
**C**	Patients eligible for device-related reimbursement	The number of patients eligible for device-related reimbursement was derived using the following formula: • = ICP-eligible and enrolled patients (from current year) * Cohort size
**D**	Annual Hospitalizations	The number of annual readmissions was derived using the following formula: • = ICP-eligible and enrolled patients (from previous year) * 31–90 Day Hospitalization Rate [See Budget Impact Model Values & References]
**E**	Hospital Reimbursement	Hospitals: • CMS Reimbursement for ICP and ICP + NIV (AVAPS-AE (Trilogy 100)) includes reimbursement for hospital admission [15] [See Budget Impact Model Values & References] Payers: • CMS Reimbursement for hospital admissions (including 0–30 day) was derived using the following formula: • = (100%—% of hospital readmissions within 0–30 days [5,8,12]) * CMS Reimbursement amount for hospital admissions [See Budget Impact Model Values & References]
**F**	Device Reimbursement	CMS Reimbursement for patients receiving NIV (AVAPS-AE (Trilogy 100)) includes a combined reimbursement amount for device acquisition, supplies and supportive care [See Budget Impact Model Values & References]
**G**	30-Day Readmission Cost	Total hospital readmission cost was derived using the following formula: • = Monthly readmissions * Cost of hospital readmissions (See 'Model Values and References' tab for details) [See Budget Impact Model Values & References]
**H**	31–90 Day Hospitalization Cost	Total 90-day hospitalization cost was derived using the following formula: • = 90 Day Hospitalizations * Cost of hospitalizations (All-cause) [See Budget Impact Model Values & References]
**I**	Device Acquisition/ Repair Cost	Total device acquisition cost was derived using the following formula: • = Number of COPD patients prescribed NIV * Cost of NIV (AVAPS-AE (Trilogy 100)) acquisition [See Budget Impact Model Values & References] Total device repair cost was derived using the following formula: • = Patients eligible for device-related reimbursement (from previous year) * Cost of NIV (AVAPS-AE (Trilogy 100)) repair/replacement [See Budget Impact Model Values & References]
**J**	Supplies	Supplies include accessories and consumables such as filters, tubing, circuits, masks, water chamber etc. See 'Model Values and References' tab for detailed list/costs. Total supplies cost was derived using the following formula: • = Patients eligible for device-related reimbursement * Cost of NIV (AVAPS-AE (Trilogy 100)) supplies, included at a frequency that is clinically reasonable [See Budget Impact Model Values & References]
**K**	Supportive Care (i.e. Respiratory Therapy)	Supportive care includes home medical equipment provider (HME) support services as part of home management program such as visits from a respiratory therapist (RT). Supportive care costs are included for NIV (AVAPS-AE (Trilogy 100)) [See Budget Impact Model Values & References]
**L**	Set-up / Other	Total set-up cost was derived using the following formula: • = Number of COPD patients qualified for NIV * NIV (AVAPS-AE (Trilogy 100)) set-up labor costs [See Budget Impact Model Values & References]

**Table 3:** Budget impact model values & references.

Value	Description	Reference
**21.0%**	30-Day readmission rate for the overall patient cohort in a 3-month timeframe preceding ICP implementation (Pre-ICP)	Alabama Integrated Care Program. 2016–2018
**61.0%**	31–90 Day hospitalization rate for the overall patient cohort in a 3-month timeframe preceding ICP implementation (Pre-ICP)	Alabama Integrated Care Program. 2016–2018
**16.0%**	30-Day readmission rate in a 3-month timeframe preceding ICP implementation for a patient cohort who enrolled in ICP that were not prescribed NIV (ICP)	Alabama Integrated Care Program. 2016–2018
**11.0%**	30-Day readmission rate for an ICP patient cohort after ICP implementation	Alabama Integrated Care Program. 2016–2018
**111.4%**	31–90 Day hospitalization rate in a 3-month timeframe preceding ICP implementation for a patient cohort who enrolled in ICP that were not prescribed NIV (ICP)	Alabama Integrated Care Program. 2016–2018
**0.0%**	30-Day readmission rate in a 3-month timeframe preceding ICP implementation for a patient cohort who enrolled in ICP that were prescribed NIV (ICP + NIV)	Alabama Integrated Care Program. 2016–2018
**6.3%**	30-Day readmission rate for an ICP + NIV patient cohort after ICP implementation	Alabama Integrated Care Program. 2016–2018
**100.0%**	31–90 Day hospitalization rate in a 3-month timeframe preceding ICP implementation for a patient cohort who enrolled in ICP that were prescribed NIV (ICP + NIV)	Alabama Integrated Care Program. 2016–2018
**9.1%**	31–90 Day hospitalization rate for an ICP + NIV patient cohort after ICP implementation	Alabama Integrated Care Program. 2016–2018
**10.2%**	Mortality rate of all patients after enrolling in the ICP	Alabama Integrated Care Program. 2016–2018
**$6,971**	CMS Reimbursement to a US hospital for a patient admission (excluding 0-30-day readmissions)	CMS "Medicare charge in-patient summary" for DRG 190 with MCC FY2016 within national summary FY2016 (Updated August 2018)
**$1,057**	CMS Reimbursement to home medical equipment provider (HME) for NIV (AVAPS-AE (Trilogy 100)) device, supplies and supportive care (per month)	2019 CMS Average. CPT code E0465, E0466
**$11,400**	Cost per Readmission (within 30-days of initial admission)	American Hospital Network, 2016 Assume ~8% yearly increase
**$10,862**	Cost per Hospitalization (All-cause admission)	American Hospital Network, 2016 Assume ~8% yearly increase
**$17,600**	Cost to HME of NIV (AVAPS-AE (Trilogy 100)) acquisition	Philips-reported repair rates (obtained, 2018)
**$14.74**	Cost to HME of NIV (AVAPS-AE (Trilogy 100)) repair Default value of $13.90 was obtained using the following formula: 10.5% of the devices require some type of repair/service (after year 2) * $132/avg repair cost per unit (inflated to 2018 values)	AVAPS-AE (Trilogy 100) Supplies MSRP (obtained, 2018)
**$1,036**	Cost to HME of NIV (AVAPS-AE (Trilogy 100)) supplies (annual) Filters, bacteria (22mm), single use 50-pack (MSRP $160.00) at 2 packs used per year = $320.00 per year; Disposable circuits (adult non-invasive passive, disposable, non-heated 10-pack) (MSRP $83.80) at 2 packs per year = $167.60 per year; NIV mask (MSRP $274) at 2 per year = $548.00. Annual TOTAL supplies costs = $320.00 + $167.60 + $548 = $1,035.60	• RT site visits ($75 per visit): 5 visits in first month; one visit per month for next 11 months • Phone call to patient ($15 per call): 5 min average plus 15 min for documentation; 6 calls per year
**$1,290**	Annual cost to HME of NIV (AVAPS-AE (Trilogy 100)) supportive care	1.5 hour for initial setup plus mileage and documentation

### Base case and scenario analyses

The base case was defined as 1,033 patients diagnosed with COPD. Scenarios examined cumulative savings during years 0 to 5 for payers covering 5,000, 10,000, 25,000, and 50,000 patients enrolled in the ICP.

### Sensitivity analyses

Probabilistic sensitivity analysis was performed for individually varying model parameters using reasonable lower and upper bounds for each parameter. Normal distribution was applied to readmission and hospitalization rates. Lower and upper bounds for costs and reimbursements were based on published values. When appropriate parameters were not found, model inputs were varied by ±50% to capture the broad variance of several parameters such as hospital costs. Sensitivity analysis was performed using @RISK software (Palisade Corp., Ithaca, NY) in the advanced sensitivity analysis mode.

## Results

Implementing this initiative across multiple hospitals was considered an initial point of success. However, the question remained, “Can integrated care for COPD patients yield improved clinical and economic outcomes?”. To explore the effect on the Integrated Care Pathway (ICP), we retrospectively analyzed 30-day COPD readmissions, and 90-day all-cause hospitalizations and modeled economic outcomes based on CMS-published values for hospitalization and readmission costs and reimbursements. Over 9 months lowered both 30-day readmissions and rolling 90-day all-cause hospitalizations. For patients receiving ICP care, 30-day readmissions declined by 57%, and 90-day all-cause hospitalizations declined by 36%. Calculated total direct cost savings per patient per year (PPPY) for the hospital were $29,044 and $21,470 for years one and five, respectively. Cumulative direct savings projected over five years of ICP implementation was $129 million. Outcomes of patients who were prescribed NIV by their physician as part of their ICP care (ICP+NIV) were also analyzed. This subset of patients realized a 71% reduction in 30-day readmissions and an 85% reduction in 90-day all-cause hospitalizations. These outcomes were associated with year-one and year-five cost savings of $51,239 and $35,954 PPPY, respectively, compared to traditional care. Projected cumulative savings after five years exceeded $200 million.

Implementation of the ICP reduced rolling 90-day all-cause hospitalizations for 1,033 patients with COPD. Hospitalization rates decreased significantly (p < 0.0001) from 61% Pre-ICP (standard care) to 39% during the ICP program and were associated with cost savings of $11,263 per patient per year (PPPY, Table 4) in the first year, and $9,146 savings PPPY in the fifth year. Compared to Pre-ICP, 5-year cumulative savings related to ICP was $52 million. All-cause hospitalizations declined to 9% for qualified patients who were prescribed NIV as part of the ICP (ICP+NIV) resulting in cost savings relative to Pre-ICP of $20,535 and $15,138 PPPY in the first and fifth year, respectively, and cumulative savings of $91 million after five years (Table 4).

**Table 4:** Projected outcomes from Integrated Care Pathway implementation, base case.

Pre-ICP vs ICP	Year 0	Year 1^e^	Year 2	Year 3	Year 4	Year 5
**Pre-ICP**						
Hospital Reimbursement	$0	($41,636,778)	($38,051,863)	($34,742,287)	($31,763,118)	($29,020,741)
Total Annual Cost	$0	($41,636,778)	($38,051,863)	($34,742,287)	($31,763,118)	($29,020,741)
**ICP only**						
Hospital Reimbursement	$0	($30,001,784)	($26,955,697)	($24,226,007)	($21,781,692)	($19,573,124)
Total Annual Cost	$0	($30,001,784)	($26,955,697)	($24,226,007)	($21,781,692)	($19,573,124)
**Pre-ICP vs ICP only**						
***Savings per Patient***	**$0**	**$11,263**	**$10,742**	**$10,180**	**$9,663**	**$9,146**
***Total Annual Savings***^***a***^	**$0**	**$11,634,994**	**$11,096,166**	**$10,516,280**	**$9,981,426**	**$9,447,616**
***Cumulative Annual Savings***^***c***^	**$0**	**$11,634,994**	**$22,731,159**	**$33,247,440**	**$43,228,865**	**$52,676,482**
**Pre-ICP vs ICP + NIV**						
**Pre-ICP**						
Hospital Reimbursement	$0	($41,636,778)	($38,051,863)	($34,742,287)	($31,763,118)	($29,020,741)
Total Annual Cost	$0	($41,636,778)	($38,051,863)	($34,742,287)	($31,763,118)	($29,020,741)
**ICP + NIV**	** **	** **	** **	** **	** **	** **
Hospital Reimbursement	$0	($7,321,768)	($6,577,182)	($5,910,973)	($5,316,610)	($4,774,498)
Device Reimbursement^d^	$0	($13,102,572)	($11,770,752)	($10,578,456)	($9,513,000)	($8,549,016)
Total Annual Cost	$0	($20,424,340)	($18,347,934)	($16,489,429)	($14,829,610)	($13,323,514)
**Pre-ICP vs ICP + NIV**						
***Savings per Patient***	**$0**	**$20,535**	**$19,074**	**$17,670**	**$16,393**	**$15,196**
***Total Annual Savings***^***b***^	**$0**	**$21,212,438**	**$19,703,929**	**$18,252,858**	**$16,933,508**	**$15,697,226**
***Cumulative Annual Savings***	**$0**	**$21,212,438**	**$40,916,367**	**$59,169,225**	**$76,102,733**	**$91,799,959**

ICP, Integrated Care Pathway; NIV, Noninvasive Ventilation

^a^ Total Annual Cost (ICP)–Total Annual Cost (Pre-ICP)

^b^ Total Annual Cost (ICP+NIV)–Total Annual Cost (Pre-ICP standard care)

^c^ Sum of cost savings for present and all preceding time frames examined

^d^ Represents combined device, supplies, and supportive care reimbursement

^e^ Year one outcomes are derived from the ICP; all subsequent yearly outcomes are simulated

**Fig 3:**
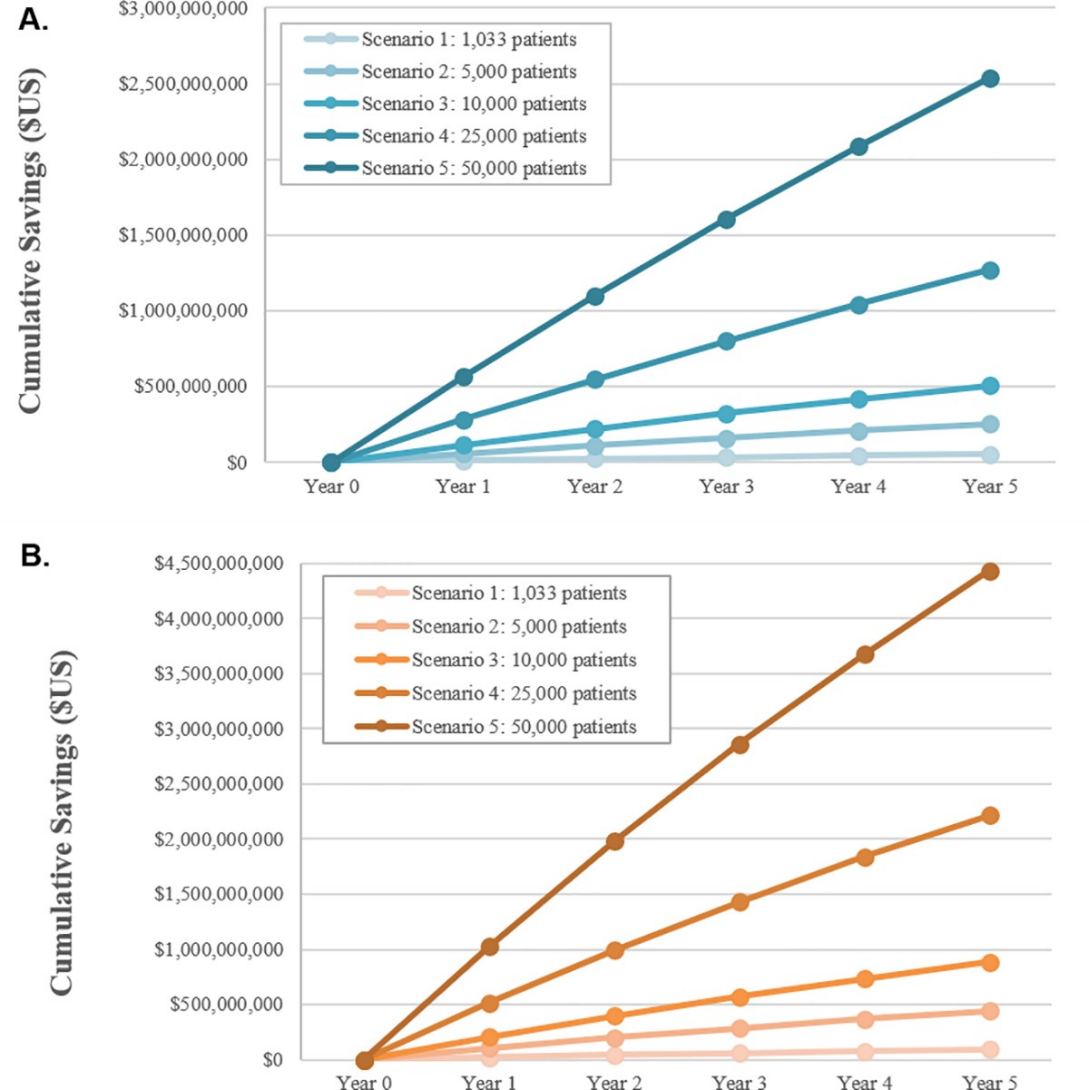
Cumulative savings scenario analyses. Scenario analyses examined cumulative savings associated with varying number of patients with COPD (all other base-case parameters constant). A. Cumulative savings when comparing Pre-ICP outcomes to ICP outcomes. B. Cumulative savings when comparing Pre-ICP outcomes to ICP + NIV outcomes.

Scenario analysis of economic outcomes associated with ICP implementation compared to Pre-ICP standard care COPD care was undertaken to investigate the effect of different population sizes (5,000, 10,000, 25,000, and 50,000 patients) on annual savings over time. Results from these analyses indicate that cost savings were not attenuated by increasing populations. (See Fig 3).

**Fig 4:**
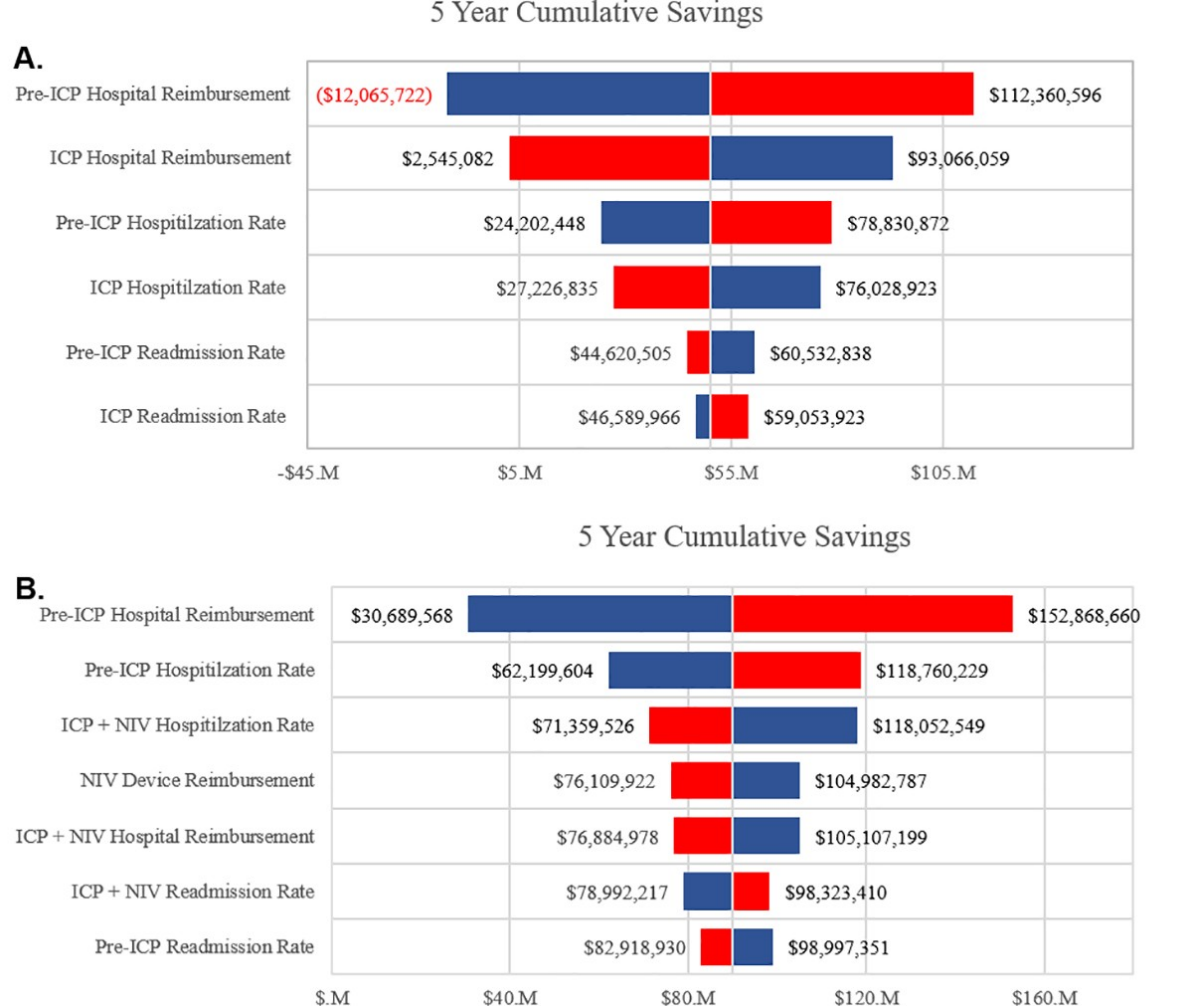
Probabilistic sensitivity analysis of cumulative savings associated with ICP implementation. Sensitivity analysis was carried out by independently modifying individual model inputs. 5-year cumulative savings, when comparing A) Pre-ICP and ICP economic outcomes and B) when comparing Pre-ICP and ICP + NIV economic outcomes. Red bars represent savings associated with an increase in the value of a given model input. Blue bars represent savings associated with a decrease in the value of a given model input. () indicates a negative number.

Probabilistic sensitivity analysis (PSA) was conducted to determine which of the model’s parameters have the largest effect on model outcomes [23], specifically 5-year cumulative cost savings. Model parameters are represented as distributions around the base-case value from which a random sampling of input values is drawn for each instance of the model being executed. This was repeated 1,000 times and the results are described in Fig 4. Sensitivity analysis of Pre-ICP vs ICP savings reveal that savings are most strongly impacted by standard care hospital reimbursement followed by ICP hospital reimbursement, standard care hospitalization rate, and ICP hospitalization rate (Fig 4A). Sensitivity analysis of Pre-ICP vs ICP+NIV savings indicate that savings are most strongly impacted by standard care hospital reimbursement, followed by standard care hospitalization rate, ICP+NIV hospitalization rate, and NIV device reimbursement (Fig 4B).

## Discussion

Integrated care has been defined as “*the continuum of patient centered services organized as a care delivery value chain for patients with chronic conditions with the goal of achieving the optimal daily functioning and health status for the individual patient and to achieve and maintain the individual’s independence and functioning in the community*” [24]. Management of patients with COPD represents a substantial financial burden to payers [25, 26]. Acute exacerbations of COPD are among the most common causes of hospitalizations for this population [3]. Studies have demonstrated that hospital readmission rate was significantly reduced by employing a multi-faceted post-acute care approach that included the use of NIV, oxygen therapy, proper medications and respiratory therapy follow-up [11]. This paper represents the first economic model of cost savings associated with the adoption of a continuous, multifaceted, quality improvement program for COPD care delivery.

Based on previously reported data [27, 28], the present analysis estimates a sizeable reduction in all-cause hospitalizations through five years, as well as considerable savings for payers in all scenarios that were examined. This analysis assumes that COPD patients represent a cost to the payer, and the cost savings do not imply positive cash flow. Rather, the budget impact is net positive because of reduced costs of care relative to more standard management models.

By substituting a previously fragmented health care model with a treatment paradigm founded on coordinating care and care transitions across the entire treatment continuum, we have shown improvements in patient outcomes and associated healthcare costs. While the four pillars addressed in this initiative are not the only aspects of COPD patient care management, we have shown that 1) these four pillars are sufficient to create meaningful improvement in patient outcomes and cost savings and 2) they can be addressed without any financial burden of hiring new staff. Additionally, although the use of NIV resulted in additional cost savings compared to both standard care and ICP alone, this does not necessarily infer a causal relationship between NIV use and accrued savings. Instead, we hypothesize that the ICP, by enabling physicians to make more informed management decisions at the appropriate time for any given patient, is the direct driver of these economic benefits.

Mortality and dropout rates were tracked during this quality improvement initiative to inform our interpretation of the above-presented event rates more clearly (i.e. hospitalizations and readmissions). While not affecting our time series analysis, significant mortality and/or dropout rates could be interpreted as a negative outcome, although the model simulations could be interpreted as a cost savings to payers. Our assessment revealed that 1) mortality observed in this initiative was within or below previously published rates [29] and importantly, 2) the mortality event rates were not influenced by the decision to prescribe NIV.

A key finding from this initiative was that developing and implementing an effective and efficient ICP program based on lean-like principles requires commitment and considerable investment of time and human resources from all stakeholders in the care pathway and workflows. It also requires an understanding of, and ongoing dedication to, lean-like principles. While there was no overhead financial cost to hospitals or payers to incorporate into a cost analysis, we appreciate that several factors may affect other institutions’ ability to implement this pathway. Given that, several key goals should be aimed for when initiating an integrated care program including increasing cross-functional coordination, discovery and identification of gaps in care, and end-to-end data collection. Additionally, centralized data collection, beyond improving patient outcomes, will also improve care efficiency by improving visibility post-discharge, allowing tracking of adherence to diagnostic guidelines, and benchmarking the successes and failures of the clinical pathways, which can serve as targets for objective improvement.

Electronic health record (EHR) usage in the home health environment, through its documented reduction of clinical documentation to final claims processing times [30], may also eliminate any potential administrative challenges to patient management and treatment. Critical to this quality improvement effort is the home medical equipment provider (HME, Med-South, Inc.) who committed to this quality improvement initiative.

Previous work has suggested that the impact of respiratory therapist-led or specialist-led home intervention is unclear [31]. Some studies have shown that individualized treatment action plans managed by specialty nurse case managers [32] or respiratory nurses [33], with telephone sessions and/or home visits, did not change exacerbation rates or health care utilization among patients with COPD. In contrast, other studies have demonstrated that more intensive home care interventions involving integrated specialist and home care nurse efforts [34] reduced hospital and emergency department admission rates. In all these cases, however, the alignment of services began only when the patient has been discharged to home care rather than across the entire acute to post-acute care continuum.

Limitations of this analysis include that this initiative was carried out in hospitals within one state, and results may not be generalizable nationwide. Also, in this analysis NIV is defined as a pressure support ventilator with volume control mode [AVAPS-AE (e.g., Trilogy) as prescribed by the physician; Healthcare Common Procedure Coding System [HCPCS] codes E0464/E0471, E0466]. Outcome rates related to the use of AVAPS-AE NIV may not reflect all home NIV devices. Additionally, the model does not address any budget impact to the HME. Lastly, the model did not fully account for differences in outcomes due to seasonality of COPD symptoms. Still, the estimated savings suggest important benefits associated with the implementation of an end-to-end patient management program for COPD patients. Future studies are needed to better understand how variations in geography, program characteristics, and treatment devices, such as NIV, affect cost savings across a range of payer systems.

## Conclusions

This analysis provides evidence for the economic benefits of integrated care workflows for COPD patients. The results presented above indicate that lean-like workflow change strategies should emphasize rapid, evidence-based assessment and diagnosis, efficient care transitions, provider alignment, respiratory therapist-led follow-up care, continuous data collection and centralization, and objective performance analysis across the care continuum. Estimated savings were primarily driven by a reduction in all-cause 90-day hospitalizations. This initiative also provides support for post-acute care adoption of EHR systems through its data transmission, centralization and aggregation paradigm. Incorporation of an integrated care model that promotes early, comprehensive diagnosis and efficient transitions of care can reduce hospitalizations and potentially reduce costs for managing COPD patients.
