# Correction: A quality improvement initiative for COPD patients: A cost analysis

**DOI:** 10.1371/journal.pone.0249844

**Published:** 2021-04-02

**Authors:** David Trout, Archita H. Bhansali, Dushon D. Riley, Fred W. Peyerl, Teofilo L. Lee-Chiong

There is an error in Table 3. The information in the Reference column in rows 15–18 is incorrect. Please see the correct Table 3 here.

**Table 3:** Budget impact model values & references.

Value	Description	Reference
**21.0%**	30-Day readmission rate for the overall patient cohort in a 3-month timeframe preceding ICP implementation (Pre-ICP)	Alabama Integrated Care Program. 2016–2018
**61.0%**	31–90 Day hospitalization rate for the overall patient cohort in a 3-month timeframe preceding ICP implementation (Pre-ICP)	Alabama Integrated Care Program. 2016–2018
**16.0%**	30-Day readmission rate in a 3-month timeframe preceding ICP implementation for a patient cohort who enrolled in ICP that were not prescribed NIV (ICP)	Alabama Integrated Care Program. 2016–2018
**11.0%**	30-Day readmission rate for an ICP patient cohort after ICP implementation	Alabama Integrated Care Program. 2016–2018
**111.4%**	31–90 Day hospitalization rate in a 3-month timeframe preceding ICP implementation for a patient cohort who enrolled in ICP that were not prescribed NIV (ICP)	Alabama Integrated Care Program. 2016–2018
**0.0%**	30-Day readmission rate in a 3-month timeframe preceding ICP implementation for a patient cohort who enrolled in ICP that were prescribed NIV (ICP + NIV)	Alabama Integrated Care Program. 2016–2018
**6.3%**	30-Day readmission rate for an ICP + NIV patient cohort after ICP implementation	Alabama Integrated Care Program. 2016–2018
**100.0%**	31–90 Day hospitalization rate in a 3-month timeframe preceding ICP implementation for a patient cohort who enrolled in ICP that were prescribed NIV (ICP + NIV)	Alabama Integrated Care Program. 2016–2018
**9.1%**	31–90 Day hospitalization rate for an ICP + NIV patient cohort after ICP implementation	Alabama Integrated Care Program. 2016–2018
**10.2%**	Mortality rate of all patients after enrolling in the ICP	Alabama Integrated Care Program. 2016–2018
**$6,971**	CMS Reimbursement to a US hospital for a patient admission (excluding 0-30-day readmissions)	CMS "Medicare charge in-patient summary" for DRG 190 with MCC FY2016 within national summary FY2016 (Updated August 2018)
**$1,057**	CMS Reimbursement to home medical equipment provider (HME) for NIV (AVAPS-AE (Trilogy 100)) device, supplies and supportive care (per month)	2019 CMS Average. CPT code E0465, E0466
**$11,400**	Cost per Readmission (within 30-days of initial admission)	American Hospital Network, 2016 Assume ~8% yearly increase
**$10,862**	Cost per Hospitalization (All-cause admission)	American Hospital Network, 2016 Assume ~8% yearly increase
**$17,600**	Cost to HME of NIV (AVAPS-AE (Trilogy 100)) acquisition	Philips Trilogy 100 MSRP
**$14.74**	Cost to HME of NIV (AVAPS-AE (Trilogy 100)) repair Default value of $13.90 was obtained using the following formula: 10.5% of the devices require some type of repair/service (after year 2) * $132/avg repair cost per unit (inflated to 2018 values)	Philips-reported repair rates (obtained, 2018)
**$1,036**	Cost to HME of NIV (AVAPS-AE (Trilogy 100)) supplies (annual) Filters, bacteria (22mm), single use 50-pack (MSRP $160.00) at 2 packs used per year = $320.00 per year; Disposable circuits (adult non-invasive passive, disposable, non-heated 10-pack) (MSRP $83.80) at 2 packs per year = $167.60 per year; NIV mask (MSRP $274) at 2 per year = $548.00. Annual TOTAL supplies costs = $320.00 + $167.60 + $548 = $1,035.60	AVAPS-AE (Trilogy 100) Supplies MSRP (obtained, 2018)
**$1,290**	Annual cost to HME of NIV (AVAPS-AE (Trilogy 100)) supportive care	• RT site visits ($75 per visit): 5 visits in first month; one visit per month for next 11 months • Phone call to patient ($15 per call): 5 min average plus 15 min for documentation; 6 calls per year • 1.5 hour for initial setup plus mileage and documentation
